# Assessing the dynamic resilience of Urban Rail Transit Networks during their evolution using a ridership-weighted network

**DOI:** 10.1371/journal.pone.0291639

**Published:** 2023-09-21

**Authors:** Tian Tian, Yichen Liang, Zhipeng Peng, Yanqiu Cheng, Kuanmin Chen

**Affiliations:** 1 College of Transportation Engineering, Chang’an University, Xi’an, Shaanxi, China; 2 School of Economics and Management, Xi’an Technological University, Xi’an, Shaanxi, China; Southwest Jiaotong University, CHINA

## Abstract

The assessment of the resilience of Urban Rail Transit Networks (URTNs) and the analysis of their evolutionary characteristics during network growth can help in the design of efficient, safe, and sustainable networks. However, there have been few studies regarding the change of resilience in long-term network development. As for the existing resilience studies, they rarely consider the entire cycle of accident occurrence and repair; furthermore, they ignore the changes in network transportation performance during emergencies. Moreover, the measurement metrics of the important nodes have not been comprehensively considered. Therefore, to remedy these deficiencies, this paper proposes a URTN dynamic resilience assessment model that integrates the entire cycle of incident occurrence and repair, and introduces the network transport effectiveness index *E*(*G*^*w*^) to quantitatively assess the network resilience. In addition, a weighted comprehensive identification method of the important nodes (the *WH* method) is proposed. The application considers the Xi’an urban rail transit network (XURTN) during 2011–2021. The obtained results identify the resilience evolutionary characteristics during network growth. And longer peripheral lines negatively affect the resilience of XURTN during both the attack and the repair processes. The central city network improves the damage index *R*_*dam*_ and the recovery index *R*_*rec*_ by up to 123.46% and 11.65%, respectively, over the overall network. In addition, the *WH* method can comprehensively and accurately identify the important nodes in the network and their evolutionary characteristics. Compared to the single-factor and topological strategies, the *R*_*dam*_ is 1.17%~178.89% smaller and the *R*_*rec*_ is 1.68%~84.81% larger under the *WH* strategy. Therefore, this method improves the accuracy of the important node identification. Overall, the insights from this study can provide practical and scientific references for the synergistic development of URTN and urban space, the enhancement of network resilience, and the protection and restoration of important nodes.

## Introduction

Due to the rapid development of urban rail transit, the reliance of cities on Urban Rail Transit Networks (URTNs) is growing, which yields in placing tremendous pressure and higher demands on such networks [1]. The failure of URTNs can have serious impacts on the urban traffic and the lives of residents [2, 3]. For instance, in 2022, an attack on the New York City subway in the US caused 39 casualties as well as the shutdown of several lines. Moreover, in 2021, a sudden equipment failure at Maigaoqiao Station at L-1 of the Nanjing subway in China led to the shutdown of 26 stations and train delays on the entire line. Thus, when an emergency occurs in a URTN, it is important to improve the security of the system by increasing the network’s resistance to failures, ensuring normal operations as much as possible, and making reasonable resource allocations [4, 5] for rapid network repair. Further, the prerequisite for improving URTN’s security consists of a reasonable assessment model, which can provide optimization objectives and theoretical support for URTN design and develop a protection and recovery strategies.

In recent years, the concept of resilience has been introduced in the research of URTNs security assessment [6, 7]; moreover, the use of a complex network theory to study its resilience has become a hot topic [8, 9]. For example, King *et al*. used the quantitative metrics approach, based on the graph theory, to study the impact of the stations on network resilience [10]. Furthermore, scholars often define URTN resilience as the ability of a network to resist, absorb external perturbations, and recover to its original state [2, 6]. They also subdivide resilience into static resilience [11] and dynamic ones [12]. In more detail, the static resilience emphasizes the ability of a network to maintain its functionalities when being damaged [13]; however, the dynamic resilience includes the entire cycle of incident occurrence and reparation [14, 15]. Therefore, Table 1 lists some representative studies of the URTN resilience, where the most current ones focus on either the attack [16, 17] or the reparation [18, 19]. For instance, Tang *et al*. used the classic time-series performance loss to define the URTNs resilience and analyzed the network serviceability under random and malicious attacks [20]. Moreover, D’Lima used the mean-reverting stochastic model to analyze the recovering process from the shock state to the equilibrium state and quantified the resilience of London Underground by calculating the speed of passenger count recovery [21]. In addition, Zhang *et al*. proposed a decision-making approach to repair sequences based on the resilience evaluation, where the average efficiency is considered as the index of the network performance [22]. To sum up, this literature review shows that most of the current studies have not adequately considered the whole process of an accident. However, only the comprehensive assessment of the dynamic resilience, over the entire cycle of an emergency, can enable appropriate measures to be taken before, during, and after [2].

**Table 1:** A review of the measurement of URTN resilience.

Process-es of resilien-ce	Modeling	URTN performance indicators	Node importance measurement	Case city	Year of network in case	Author (year)
**Attract and recover**	*G = (V*, *E*, *W)*	Resistance ability, absorption ability, recovery ability	---	Beijing	2020	Ma *et al*. (2022) [2]
	*G = (V*, *E*, *W)*	Network robustness, recovery, recovery speed, resilience performance	---	Xi’an	2020	Liu *et al*. (2022) [14]
	*G = (V*, *E*, *W)*	Network efficiency considering passenger trips and travel time	Effective path betweenness	Chengdu	2019	Chen *et al*. (2022) [15]
**Attract**	*G = (V*, *E*, *W)*	Passenger flow loss	---	Toronto	2013	Diab *et al*. (2022) [17]
	*G = (V*, *E)*	Network connectivity, *E(G)*, density	*K*, *B*	Chengdu	2021	Cheng *et al*. (2022) [23]
	*G = (V*, *E)*	Connected path loss	Connected path loss	Hong Kong	2019	Chen *et al*. (2021) [16]
	*G = (V*, *E)*	*E(G)*	*E(G)* loss	Washington, DC	2019	Saadat *et al*. (2019) [24]
	*G = (V*, *E*, *W)*	Network exposure	Loss of travel time and passenger flow	Toronto	2016	King *et al*. (2019) [10]
	*G = (V*, *E*, *W)*	Number of trains delayed and canceled	---	London	2018	Pagani *et al*. *(*2019) [13]
	*G = (V*, *E*, *W)*	Node strength loss	Loss of the number of connected nodes	London	2015	Chopra *et al*. (2016) [25]
	*G = (V*, *E*, *T)*	Passenger delay and passenger flow loss	---	Paris	2015	Adjetey-Bahun *et al*. (2016) [26]
**Recover**	*G = (V*, *E*, *W*, *T)*	Passenger travel time and proportion of unaffected passengers	Station passenger volume	Chengdu	2019	Chen *et al*. (2022) [19]
	*G = (V*, *E)*	*E(G)*	---	Nanjing	2017	Zhang *et al*. (2020) [22]
	*G = (V*, *E)*	*E(G)*	*E(G)* loss	Washington, DC	2018	Saadat *et al*. *(*2020) [27]
	*G = (V*, *E*, *W)*	Topological structure and passenger flow	*K*, the loss of reasonable passageway and passenger flow	Beijing	2018	Li *et al*. (2019) [18]
	*G = (V*, *E)*	*E(G)*	*E(G)* loss	Shanghai	2015	Zhang *et al*. (2018) [28]
	*G = (V*, *E*, *W)*	Network recovery speed	*B*, station passenger volume	Shanghai	2016	Lu *et al*. (2018) [29]

The URTN, as a complex network, has different characteristics from other transportation networks, such as the road networks, the bus networks, and the railroad networks. Therefore, when studying the resilience of URTNs, their multiple complexity characteristics should be fully integrated for analysis. Therefore, the deficiencies of current studies are analyzed from three complexity of URTNs.

First, the network evolution is complex. When new lines are created, the network structure and the ridership change; therefore, the network resilience is bound to a change accordingly. However, referring to Table 1, most contemporary studies on resilience have assessed URTNs at a specific time whereas few studies have explored the changing characteristics of resilience with long-term network growth. For example, Zhang *et al*. compared the URTNs resilience in Singapore and Chongqing, and found the different geographical distribution and configurations of the interchange stations would impact the level of system resilience [22]. As for Pan *et al*., they analyzed the effect of the circle line on the resilience of subway network, and found the loop stations had a positive effect on the improvement of network resilience and the delay of cascade failure peak [30]. Because the urban rail transit is difficult to build and the renovation cost, after completion, is huge. Therefore, studying the evolutionary characteristics of the dynamic resilience from the longitudinal perspective of network development can help managers to identify the network resilience in different periods and carry out control measures, such as the network structure optimization, the security warning, and the contingency planning, in advance.

Second, the URTNs’ purpose is to carry passenger flow that is highly complex, such as the differences in the passenger flow distribution of station and the cross-sectional and the diversity of passenger travelling options. Therefore, the variation of the network ridership is an aspect that worth attention when studying resilience. However, some studies have only analyzed the topological characteristics of URTNs to measure their resilience. Researchers often regard URTNs as unweighted networks [22, 28, 31, 32], or choose topological indexes as resilience assessment metrics [24, 27]. For example, Cheng *et al*. integrated the topological metrics, such as the connectivity, the efficiency, the density, and considered them as the network performance indicators to study its resilience under different attack patterns [23]. If the passenger flow is ignored, the real change of the network performance cannot be objectively measured [33]. Therefore, to accurately assess network resilience, it is crucial to establish a ridership-weighted network and select the network performance indicator that can reflects structural and passenger flow changes as well as passenger travel choices.

In addition, stations open to the public are the starting and ending points of all passengers’ travel and they are considered as the focus for accidents prevention and restoration. The node importance is also a reflection of the complexity of URTNs. The functions of the different nodes are nonlinear and their effects on the network are very complex. Therefore, the identification of the important nodes in the network is a necessary element for the resilience analysis [34]. Identifying the important nodes, that are most damaging and most effective for network recovery, and analyzing their evolutionary characteristics, contribute to develop the risk assessment and the protection of the nodes, thus enhancing the network resilience. Table 1 shows that most contemporary studies have analyzed the node importance using only a single dimension. The most commonly used metrics are the node centrality, including the degree, the betweenness, etc. [35–37]. Moreover, important nodes have been identified by analyzing the changes at the level of the network performance after the node destruction, such as the network efficiency loss as proposed by Saadat *et al*. [24, 27], the passenger flow loss as proposed by Hu [38], the increase of the passenger travel cost as proposed by Liu [39], and the route redundancy loss as proposed by Jing [40] as well as other indicators. However, single-factor metrics can only measure the node importance from one point of view, and the incomplete consideration of the important factors can lead to inaccurate or incomplete identification of important nodes [41]. Furthermore, if there is no verification of the effect of node sequence on network function, the validity of the identification method cannot be proved [41, 42]. Hence, it is necessary to establish a comprehensive measure to identify important nodes and to verify their effectiveness.

To address the shortcomings of the existing studies and fully integrate the complexity of URTNs, this paper proposes a dynamic resilience assessment model for analyzing the dynamic resilience of URTNs growth process. Firstly, a ridership-weighted network is established. By considering the whole cycle of event occurrence and recovery, two indexes are proposed: the damage index *R*_*dam*_ and recovery index *R*_*rec*_. Their main purpose is to quantify the dynamic resilience. By comprehensively analyzing the network topology as well as the passenger flow and the passenger travel characteristics, the network transportation efficiency *E*(*G*^*w*^) is selected as the network performance index. Moreover, a new weighted comprehensive identification method (the *WH* method) is proposed to identify the important nodes in the network; as for the effectiveness of this method, it is verified by the resilience model. Finally, the 2011–2021 Xi’an Urban Rail Transit Network (XURTN) is considered as an example to analyze the network resilience from both the horizontal and the vertical perspectives, and the evolution characteristics of network resilience and the important nodes are discussed.

The main contributions of this work lie in the following aspects:

1. By considering the whole process of accidents and introducing factors, such as the passenger flow and the passenger travel behavior characteristics, a URTN weighted dynamic resilience assessment model is established; thereby, this will bridge the gap between the network topological and static resilience models.
2. The evolutionary characteristics of resilience during network growth and a detrimental effect of long peripheral lines on the resilience are found. The reasons for the resilience variation are explained from the perspective of network structure and spatial layout, which can provide important basis and feasible suggestions for the synergistic development of URTN and urban space, the planning and layout of the network.
3. The *WH* method can comprehensively and detailed identify the important nodes and their evolutionary characteristics. The *R*_*dam*_ index, under the *WH* strategy, is 1.17%~178.89% smaller and the *R*_*rec*_ index is 1.68%~84.81% larger than those under the single-factor strategies and topological strategy. Thus, it is proved that the *WH* method improves the identification accuracy of the important nodes and can provide a basis for identifying, protecting and repairing important nodes.

To sum up, the remainder of this paper is organized as follows: The Methodology section introduces the proposed URTN dynamic resilience assessment model and the *WH* method. As for the Case Study section, it presents a case analysis of the XURTN, and examines the evolution characteristics of resilience and the important nodes, as well as the reasons of evolution. Finally, the conclusion section summarizes the proposed work and delivers some future ideas.

## Methodology

### Ridership-weighted network model

The construction of a network model, which reflects the transportation function of URTNs, is a requirement for assessing its dynamic resilience. The L-space [43] was chosen to construct the topological network *G* = (*V*, *E*), where *V* represents the node set and *E* indicates the undirected edge set. Moreover, topological networks can only show whether there is a connection relationship between nodes. Thus, the weighted network can reflect the closeness of the relationship between nodes by giving weights to the edges. In this study, a weighted network model is established using the cross-sectional passenger flow that was considered as the weight of the edges. Consequently, the directed and weighted network is represented by a graph *G*^*w*^ = (*V*, *E*^*w*^, *W*), where *E*^*w*^ represents the directed edge set ($e_{ij}^{w}$ is an ordered node pair, formed by two adjacent nodes), and *W* denotes the set of ridership weights of the directed edge $e_{ij}^{w}$, where $W=\{{pf}_{e_{ij}^{w}}(\Delta t)\}$.

To reflect the development level of URTNs, *β*, *λ*, and *α* were defined as the related indices. In more detail, *β* denotes the network complexity $\beta =\frac{M}{N}$ (1) whereas *λ* represents the network connectivity $\lambda =\frac{M}{3N-6}$ (2). Moreover, *α* denotes the availability of loops $\alpha =\frac{M-N+1}{2N-5}$ (3), reflecting therefore the ability of the network to provide alternative communication paths. The larger the values of *β*, *λ* and *α* are, the higher the level of network development.

### URTN dynamic resilience measure

#### Definition of dynamic resilience

**Fig 1:**
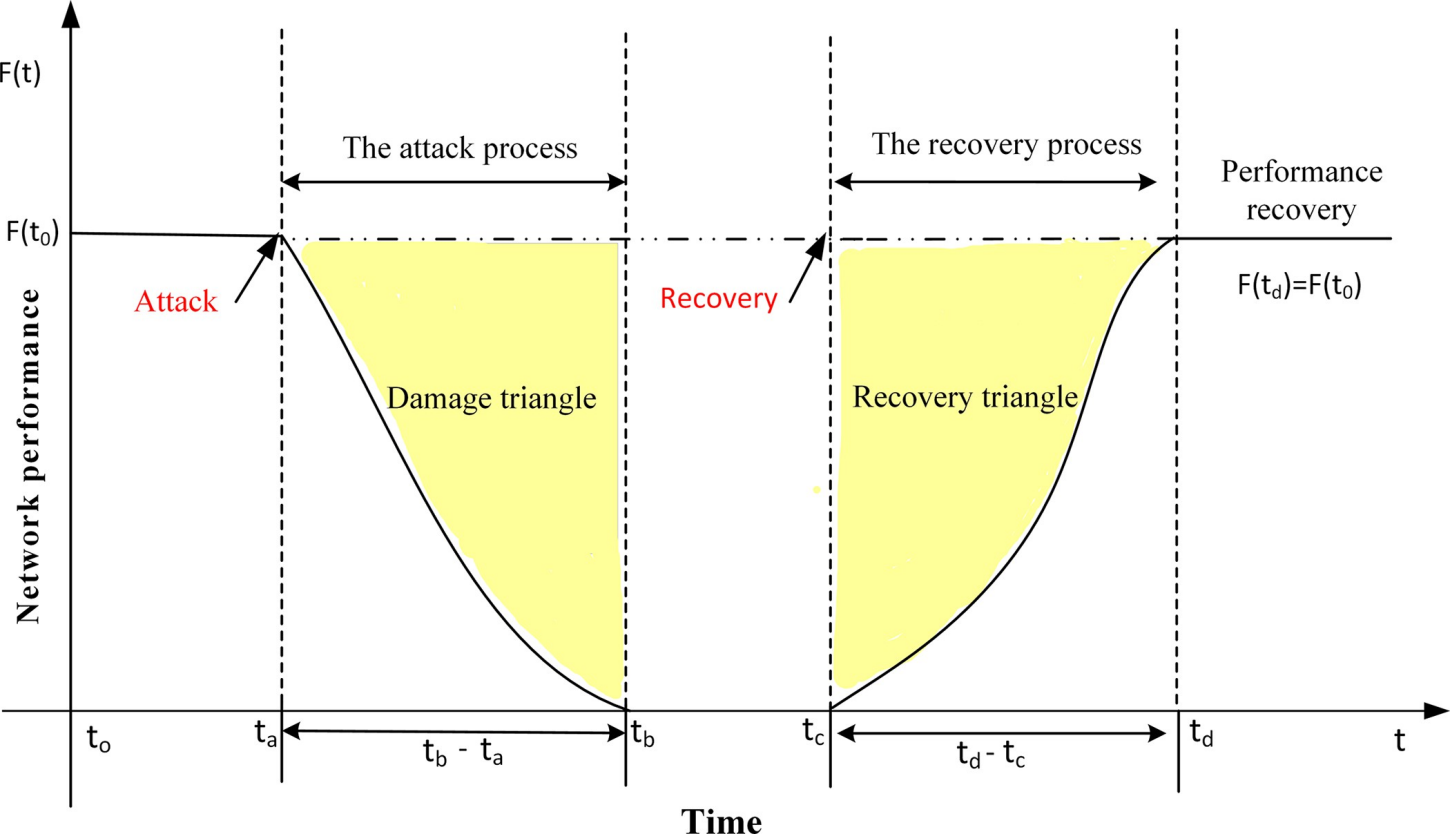
The schematic diagram of URTN dynamic resilience, damage triangle, and recovery triangle.

Most current research on URTN resilience focuses only on the attack or recovery process; however, the full cycle of accidents is rarely analyzed. Therefore, to fully reflect the connotation of dynamic resilience, *i*.*e*., attack and recovery, the URTN dynamic resilience is defined as the ability of the network to resist to disturbances and maintain transport functions during an emergency, as well as its ability to quickly recover the transport functions after appropriate reparations measurements are taken. Moreover, the URTN dynamic resilience describes the complete time-series network operation state, which is mainly divided into four processes: the initial state, the attack process, the recovery process, and the post-recovery state. Referring to Fig 1, the horizontal coordinate represents the time variable whereas the vertical coordinate *F*(*t*) shows the URTN performance. Therefore, this study focuses on the attack and recovery processes for analysis. For the implementation process, Python is used to simulate of network attack and repair processes where the stations are attack and repair objects, and each node is attacked or repaired in turn until all nodes fail or recover.

#### Two processes of dynamic resilience

The URTN attack process considers the network performance degradation after disruption. Therefore, the ability of an URTN to resist disruption and maintain a normal operation mode is analyzed by calculating the losses in the network performance under different attack strategies. As shown in Fig 1, the attack starts at *t*_*a*_. As it occurs, the network performance gradually decreases to zero. The recovery process of URTNs considers the way where the network is quickly restored to its normal functionality under the reparation measurements. It is assumed that the restoration begins after the end of the attack, *i*.*e*., the recovery measures start at *t*_*c*_. The network performance is restored to its initial normal operating level at *t*_*d*_. It is assumed that no new attacks occur during the recovery process.

Previously, Brunneau first proposed the concept of “resilience triangles”, which quantify resilience using the ratio of the area under the network performance curve after being attacked over the area under the normal network performance curve [44]. However, in this study, the modified “damage triangle” and “recovery triangle” are used to quantify the URTN resilience, which are represented by the yellow triangles in Fig 1. The quantification index of the network damage represents the damage index *R*_*dam*_, which is calculated using Eq (4), and the *F*(*t*) curve that is required to be continuous and integrable. Furthermore, the smaller the *R*_*dam*_ value is, the greater the damage to the network will be. As for the network recovery capability, it is quantified by the recovery index *R*_*rec*_, that is given by Eq (5). The larger the *R*_*rec*_ is, the faster the network restoration and the better the recovery strategy will be.


$$R_{dam}=\frac{\int_{t_{a}}^{t_{b}}F(t)dt}{F(t_{0})\cdot (t_{b}-t_{a})}$$ (4)

$$R_{rec}=\frac{\int_{t_{c}}^{t_{d}}F(t)dt}{F(t_{0})\cdot (t_{d}-t_{c})}$$ (5)

#### URTN performance indicator: Transport efficiency

To fully reflect the transportation function of a URTN, the topological connectivity, the passenger demand, and the passenger travel behavior choices are aggregated to calculate the network performance index. The network transportation efficiency *E*(*G*^*w*^) is proposed based on efficiency *E*(*G*), as proposed in Eq (6). The shorter the average path length *d*_*ij*_ and the higher the passenger flow *Q*_*ij*_ are, the higher the transportation efficiency will be.


$$E(G^{w})=\frac{1}{N(N‐1)}\sum_{i\neq j}\frac{1}{d_{ij}}*\sum_{i\neq j}Q_{ij}$$ (6)

Furthermore, the rate of change of *E*(*G*^*w*^) is considered as the network performance function *F*(*t*), and it is given by Eq (7).

$$F(t)=\frac{E(G_{t}^{w})}{E(G_{0}^{w})}$$ (7)

where *Q*_*ij*_ denotes the Original Destination (OD) passenger flow between *i* and *j*. In this study, *Q*_*ij*_ at *t*_0_ is the actual operating passenger flow of the Auto Fare Collection (AFC) system in the normal operating state. Therefore, *F*(*t*_0_) = *F*(*t*_*a*_) = *F*(*t*_*d*_) = 1 where the network operates normally and *F*(*t*_*b*_) = *F*(*t*_*c*_) = 0 where the network is completely destroyed.

It is well known that, when the network structure changes, the passenger flow in the network varies as well. In the following, the principle of OD passenger flow update is introduced when the network structure varies. To simplify the operation, the following assumptions are considered:

1. When a node is attacked or repaired, it is assumed that this node and its connected edges are removed or repaired instantaneously;
2. When a node is attacked, all trips, initially starting or ending at this station, are cancelled, and the OD traffic at this node is not transferred to its neighboring stations;
3. All stations have the function of turning back;
4. Do not consider the transportation capacity limits of the line;
5. The shortest path between two stations is identified as the original path of the passenger travel.

**Fig 2:**
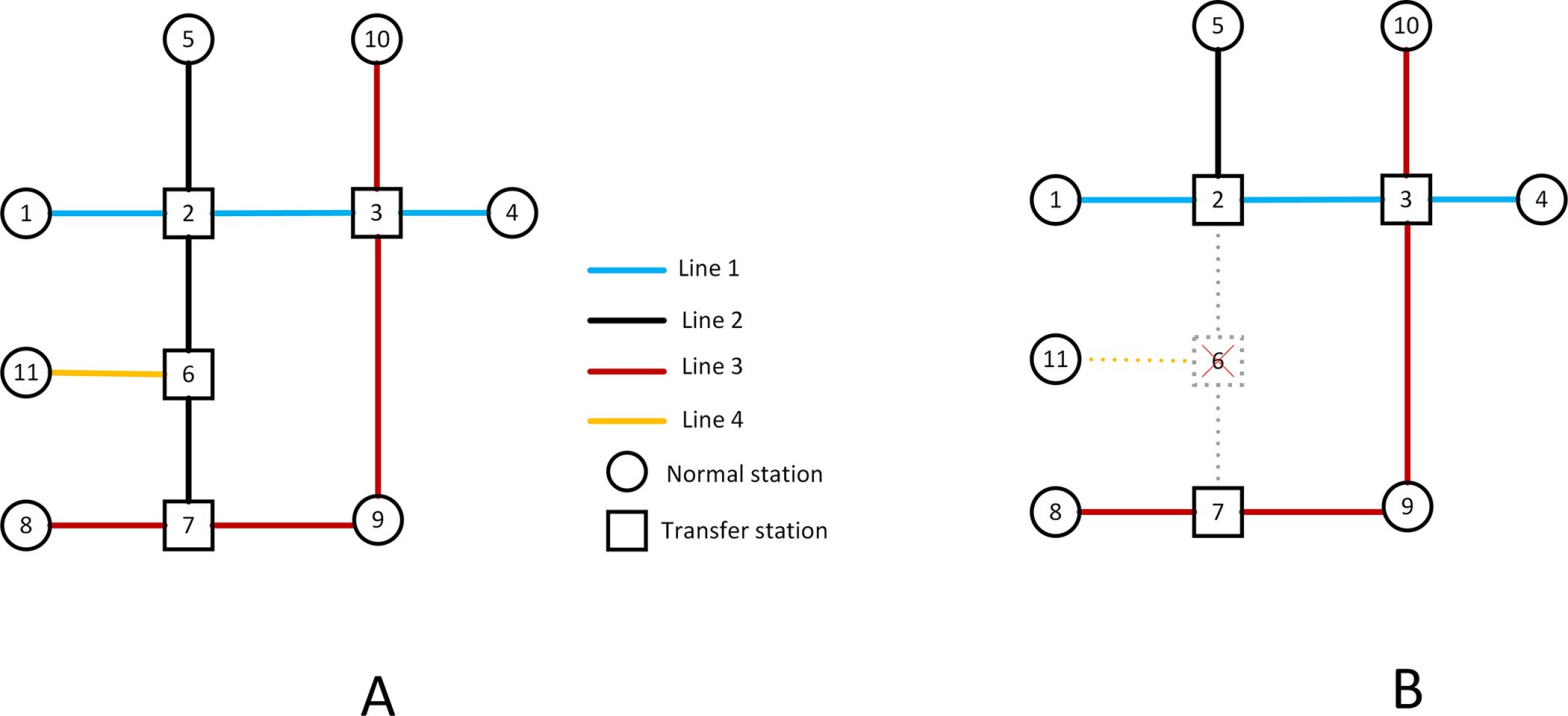
Schematic diagram of node failure.

Based on these assumptions, the network attack process is analyzed. As shown in Fig 2, when node #6 is attacked, the OD demand in the network may occur in the following situations:

1. Demand that the original travel path is not affected, *e*.*g*., the OD pair (#5, #1), then this part of the OD passenger flow remains unchanged;
2. Demand with no passable path, *e*.*g*., the OD pair (#5, #11), then this OD demand is lost;
3. The original path is broken; however, alternative paths exist to reach the destination. For example, for the OD pair (#5, # 8), the original path #5-#2-#6-#7-#8 is not available, but there are alternative paths as #5-#2-#3-#9-#7-#8. Yet, it is obvious that the alternative paths increase the travel cost of passengers. In this paper, the number of intervals on the path is chosen to quantify the travel cost, and the variable *Ω* is defined as the ratio of the number of tolerable intervals of the alternative route over the number of intervals of the original trip. Based on previous survey results, *Ω* = 1.5 is considered [45]. That is, if the ratio of the interval number between the alternative path and the original path is less than or equal to 1.5, the OD passenger flow is retained, otherwise the OD is lost.

As a result, the *Q*_*ij*_ can be calculated at each instant, including the passenger flow of the original travel path without disturbance and of the alternative path where a tolerable length exists. The next step consists of calculating the *E*(*G*^*w*^) value.

Similarly, in the recovery process, when comparing the network after each node is recovered with the original network at *t*_0_, the OD passenger flow is restored according to the connectable paths in the network. Therefore, the *Q*_*ij*_ and *E*(*G*^*w*^) values of the current network can be calculated.

### Node attack and recovery simulation

The next phase consists of simulating both processes of URTNs dynamic resilience where the nodes represent the objects of the attack and the repair. Due to the complexity of URTNs that determines their obvious heterogeneity, a few important nodes occupy a dominant position in the network. Therefore, the first task is to identify the important nodes and find the sequence of nodes that has the greatest impact on the network resilience.

#### Important node identification

Different indicators measure the importance of the nodes from different perspectives. The incomplete consideration of factors affecting the node importance will lead to inaccurate or incomplete identification of the important nodes. Therefore, the node centrality and performance are aggregated to measure the node importance from multiple perspectives. The node centrality indexes, closely related to URTN topology and passenger flow, were selected, namely *K*_*i*_, *S*_*i*_, *B*_*i*_, *WB*_*i*_, *C*_*i*_, and *WC*_*i*_. Then, by calculating the failure impact of a single node on the network performance, the importance of the node is determined. The node connectivity performance *P*_*i*_ and transportation performance *F*_*i*_ are selected to analyze the changes of the topological connectivity and the passenger flow after the node failure. The definitions and calculation formulas of these indicators are proposed in Table 2.

**Table 2:** The definitions and formulas for node centrality and performance indicators.

	Index	Formula	Definition
**Node centrality**	Degree *K*_*i*_	$K_{i}=\sum_{j=1}^{N}a_{ij}$ (8)	*K*_*i*_ is the number of connected edges of *i*.
	Strength *S*_*i*_	$S_{i}=\sum_{j\in V_{i}}{\mathrm{pf}}_{e_{\mathrm{ij}}}(\Delta t)$ (9)	*S*_*i*_ represents the total weights on the directed edge between *i* and the adjacent nodes.
	Betweenness *B*_*i*_	$B_{i}=\frac{\sum_{o\neq d\neq i}n_{od}^{'}}{\sum_{o\neq d}n_{od}},(o,d\in V)$(10)	*B*_*i*_ is the ratio of the number of shortest paths passing through *i* to the number of shortest paths between all pairs of nodes.
	Weighted betweenness *WB*_*i*_	$WB_{i}=\frac{\sum_{l=1}^{n^{i}}\sum_{x\in l}w_{l}^{i,x}}{\sum_{l=1}^{n}\sum_{x\in l}w_{l}^{x}}$ (11)	*WB*_*i*_ is the ratio of the sum of weights on all shortest paths passing through *i* to the sum of weights on all shortest paths in the entire network.
	Closeness *C*_*i*_	$C_{i}=(N‐1)*{\left[\sum_{j=1}^{N}d_{ij}\right]}^{-1}$ (12)	*C*_*i*_ describes the ability of the node to influence other nodes in topological network.
	Weighted closeness *WC*_*i*_	$WC_{i}=(N‐1)*{\left[\sum_{j=1}^{N}d_{{}_{ij}}^{w}\right]}^{-1}$ (13)	*WC*_*i*_ describes the power of node over other nodes under the action of the weight.
**Node performance**	Connectivity performance *P*_*i*_	$P_{i}=1-\frac{PG'}{PG}$ (14)	*P*_*i*_ reflects the change in the number of connectable node pairs after *i* is attacked. If the network connectivity is significantly reduced, node is more important.
	Transportation performance *F*_*i*_	$F_{i}=1-\frac{F_{i}^{'}}{F}$ (15)	*F*_*i*_ reflects the change of passenger flow after *i* is attacked. The greater the *F*_*i*_, the more important node *i*. Where *F* denotes the sum of all cross-sectional flows in the network during normal operation. After the node failure, the network ridership is updated according to OD passenger flow update principle proposed above, and then assigned using all-or-nothing method. So, the $F_{i}^{\prime }$ can be calculated by the sum of all cross-sectional flows after the node *i* fails.

Based on the single-dimension indices described previously, the concept of the comprehensive importance of the nodes is introduced. The weighted comprehensive importance of nodes *WH*_*i*_ is quantified by the sum of *K*_*i*_, *S*_*i*_, *B*_*i*_, *WB*_*i*_, *C*_*i*_, *WC*_*i*_, *P*_*i*_, and *F*_*i*_ multiplied by their weighted coefficients, as given by Eq (16). The unweighted comprehensive importance of *H*_*i*_ is calculated using Eq (17). In this study, the Principal Component Analysis (PCA) is chosen to determine the weighted coefficients *μ* and *δ*. Moreover, the previously described node centrality and performance indexes are positively correlated with node importance, which ensures the effectiveness of the PCA method.


$$WH_{i}=\mu_{1}*K_{i}+\mu_{2}*S_{i}+\mu_{3}*B_{i}+\mu_{4}*WB_{i}+\mu_{5}*C_{i}+\mu_{6}*WC_{i}+\mu_{7}*P_{i}+\mu_{8}*F_{i}$$ (16)

$$H_{i}=\delta_{1}*K_{i}+\delta_{2}*B_{i}+\delta_{3}*C_{i}+\delta_{4}*P_{i}$$ (17)

**Node attack and recovery strategy.** In this study, random and malicious attack and recovery strategies were adopted for simulation. The specific strategies are defined as follows:

1. *Random strategy*: Many emergencies, encountered in URTNs, can be regarded as random, such as the equipment failure and the natural disasters; moreover, random events occur with the same probability at every station. The nodes are attacked and recovered in sequence according to the random order of the node ID;
2. *Malicious strategy*: Some terrorist attacks, passenger flow congestion, and other events can be regarded as malicious attacks on the nodes when the attack is purposefully carried out on important stations. Several research results show that the attack strategy, with the maximum betweenness of nodes, will lead to a fast network destruction [13, 45, 46]. Therefore, the nodes are attacked and recovered in in order of *B*_*i*_, *WB*_*i*_, *H*_*i*_ and *WH*_*i*_ from largest to smallest, *i*.*e*., *B max*, *WB max*, *H max*, and *WH max*.

## Case study

### Basic information of the Xi ’an urban rail transit network

**Fig 3:**
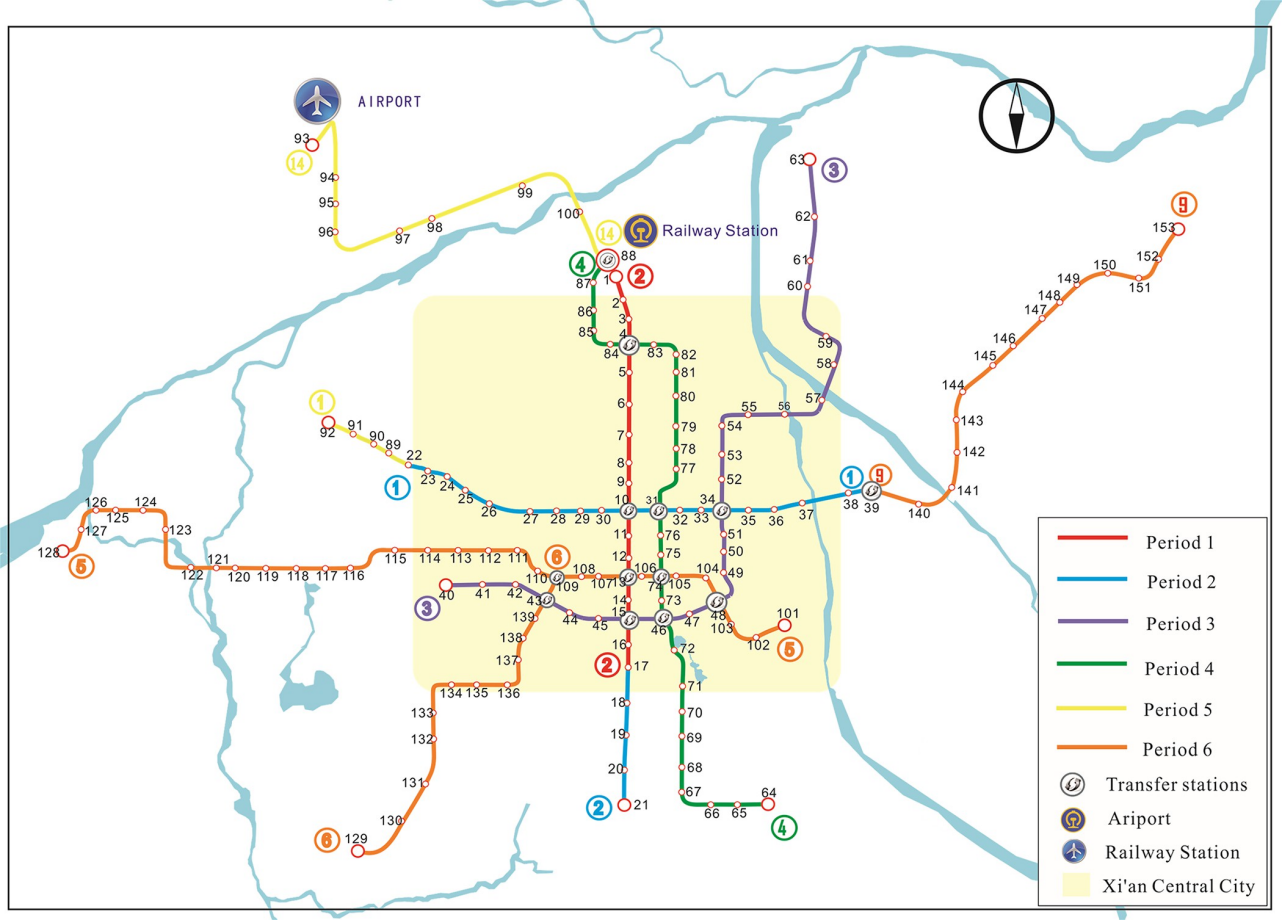
The spatiotemporal expansion of the 2011–2021 XURTN.

Xi’an is in a flat, intact region of Shaanxi Province, China, and its core area is less disturbed by the natural geographical conditions. The XURTN has been open and operating for more than ten years (2011 to the present). To study its resilience evolution characteristics, the XURTN was divided into six periods according to its development process, and the line opening information is presented in Fig 3 and Table 3. It can be seen that from 2011 to 2021, XURTN experienced the process of the construction of central-city lines and the outward expansion of the network, which represents the development process that many URTN may experience. Therefore, the XURTN can be used as a typical case to study the network evolution.

**Table 3:** The evolutionary processes of the XURTN.

Period	Network characteristics	Opening lines	Opening time	Passenger flow sampling date
**1**	Single-line operation	The first phase of *L-2*	2011.11	2012.03
**2**	"Cross" operation	The first phase of *L-1* The second phase of *L-2*	2013.9 2014.6	2014.12
**3**	Opening of the L-shaped line within the central city	*L-3*	2016.11	2017.06
**4**	Opening of the parallel line within the central city, share the north-south passenger flow	*L-4*	2018.12	2019.06
**5**	Opening of the outer extended line	The second phase of *L-1* Airport Line *L-14*	2019.9	2020.11
**6**	Three lines are opened, in which L-5 is the encrypted line in central city, L-6 and L-9 are the two outer extension lines.	*L-5* *L-6* *L-9*	2020.12	2021.04

The next phase consisted of setting up the ridership-weighted network models for the six periods of the XURTN. First, 153 stations of the XURTN were coded according to the order in which they were opened. Then, the passenger flow data from 2011 to 2021 was collected from the Xi’an urban rail transit AFC system. The passenger flow sampling dates were selected as about six months after the lines opening, as shown in Table 3. These dates were considered fairly regular without the Chinese public holidays, winter/summer holidays, or the effects of the COVID-19 pandemic. Therefore, the XURTN ridership-weighted network models were established with the average daily cross-sectional passenger flow as their edge weights.

Next, the basic indicators of the six periods were analyzed, as shown in Table 4. The network scale and ridership have continued to grow. Based on the *β*, *α*, and *γ* values presented, in the first two periods of the XURTN, where *β* < 1, *α* = 0, the network structure was too simple. From the period 3, *β* and *α* gradually increased, indicating the emergence of the loop structure in the network. Moreover, the *γ* value was found to have a U-shaped trend, and decreased to its lowest value in period 2. Starting from period 3, *γ* gradually increased. It can be known that the Period 3 is the starting point of XURTN entering the network stage where the network skeleton was basically formed. It is noteworthy that the *β*, *α*, and *γ* all decreased in period 5, which shows that the structure of the network was negatively affected by the too-long extension lines.

**Table 4:** The basic parameters of the XURTN in six periods.

Period	1	2	3	4	5	6
***N***_***L***_	1	2	3	4	5	8
***L***	20.5	52.5	91.65	126.85	162.26	244.69
***N***	17	39	63	88	100	153
***M***	16	38	63	90	102	158
***N***_***C***_	0	1	3	6	7	13
***N***_***P***_	0	0.0256	0.0462	0.0682	0.0700	0.0850
***PF* [×10** ^**4**^ **]**	14.76	93.91	160.81	253.70	262.40	427.06
***β***	0.9412	0.9744	1	1.0227	1.0200	1.0327
***α***	0	0	0.0083	0.0175	0.0154	0.0199
**γ**	0.3556	0.3423	0.3443	0.3487	0.3469	0.3489

**Fig 4:**
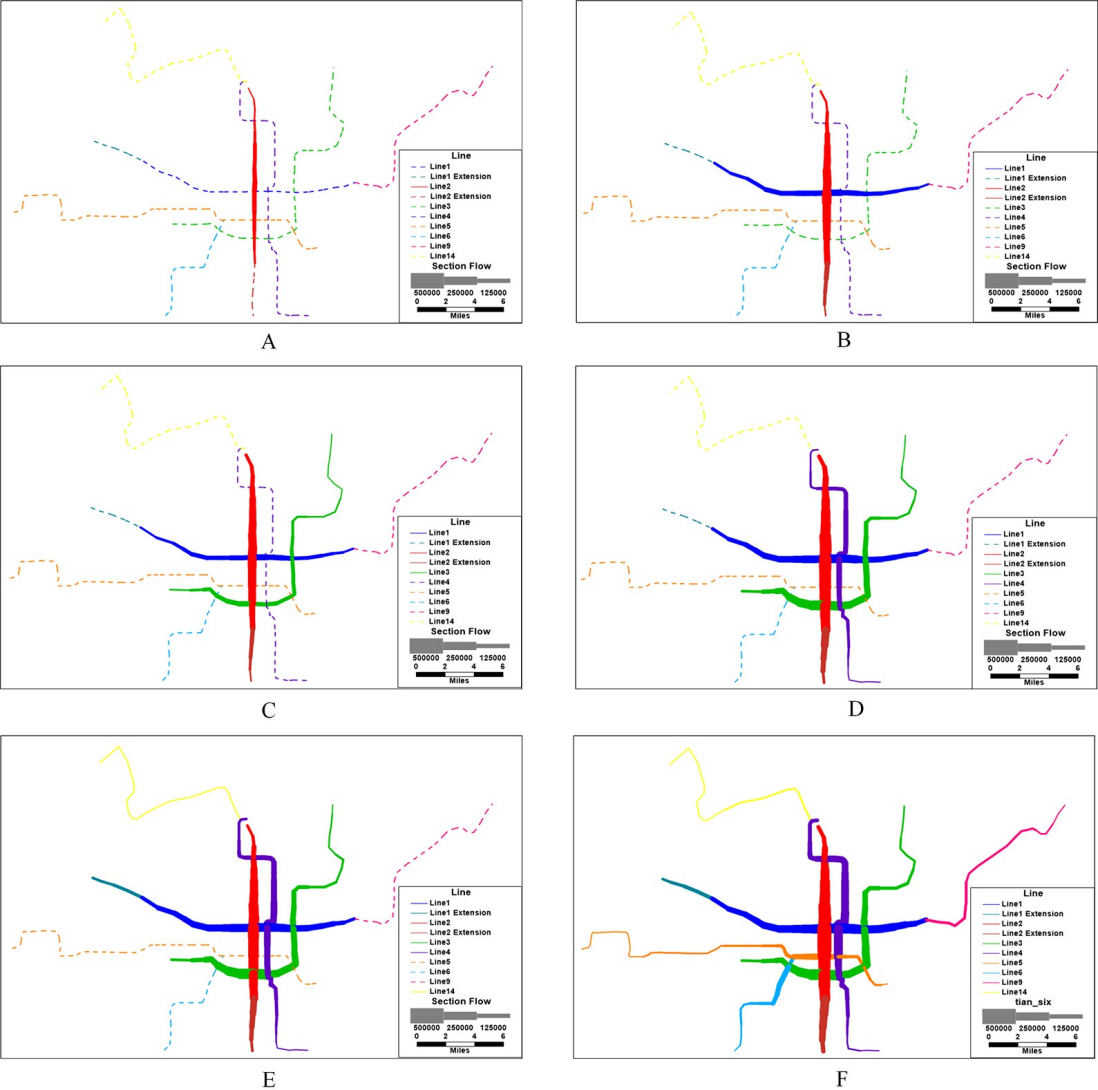
The cross-sectional passenger flow distribution map in six periods. A: Period 1, B: Period 2, C: Period 3, D: Period 4, E: Period 5, F: Period 6.

To sum up, XURTN has formed a single-center network structure. The central network has dense lines, diverse routes, and large passenger flows (the cross-sectional passenger flows is shown in Fig 4). Moreover, the outer lines extend to different areas and the regional accessibility is gradually improving, especially the outer areas of the city are covered. However, there exist several peripheral lines of long lengths and most of them have only one interchange station with the central network. The level of the direct access between the different lines is low, and the connectivity and accessibility of the network are not evenly distributed between the central city and the peripheral areas.

### Identification of the important nodes during network evolution

Based on the XURTN modeling, the dynamic resilience was studied under the node attack and recovery scenarios. The first task consisted of identifying the important nodes to find the node sequences having the greatest impact on the URTN resilience. From the above analysis, the lines in the first two periods are too simple, and the networked features are not obvious. The period 3 presents the turning point for XURTN to enter the networked stage, so the next analysis starts from the period 3.

Firstly, the centrality and performance of each node were calculated, then the PCA method was adopted. Both Kaiser–Meyer–Olkin test and Bartlett test were then applied, and the results show that the *KMO-value* is greater than 0.7 whereas the *P-value* is smaller than 0.001. This demonstrates that the data is suitable for PCA, and that the principal components can explain the information to a high degree. Then, the *WH*_*i*_ and the *H*_*i*_ of the nodes were subsequently calculated.

**Fig 5:**
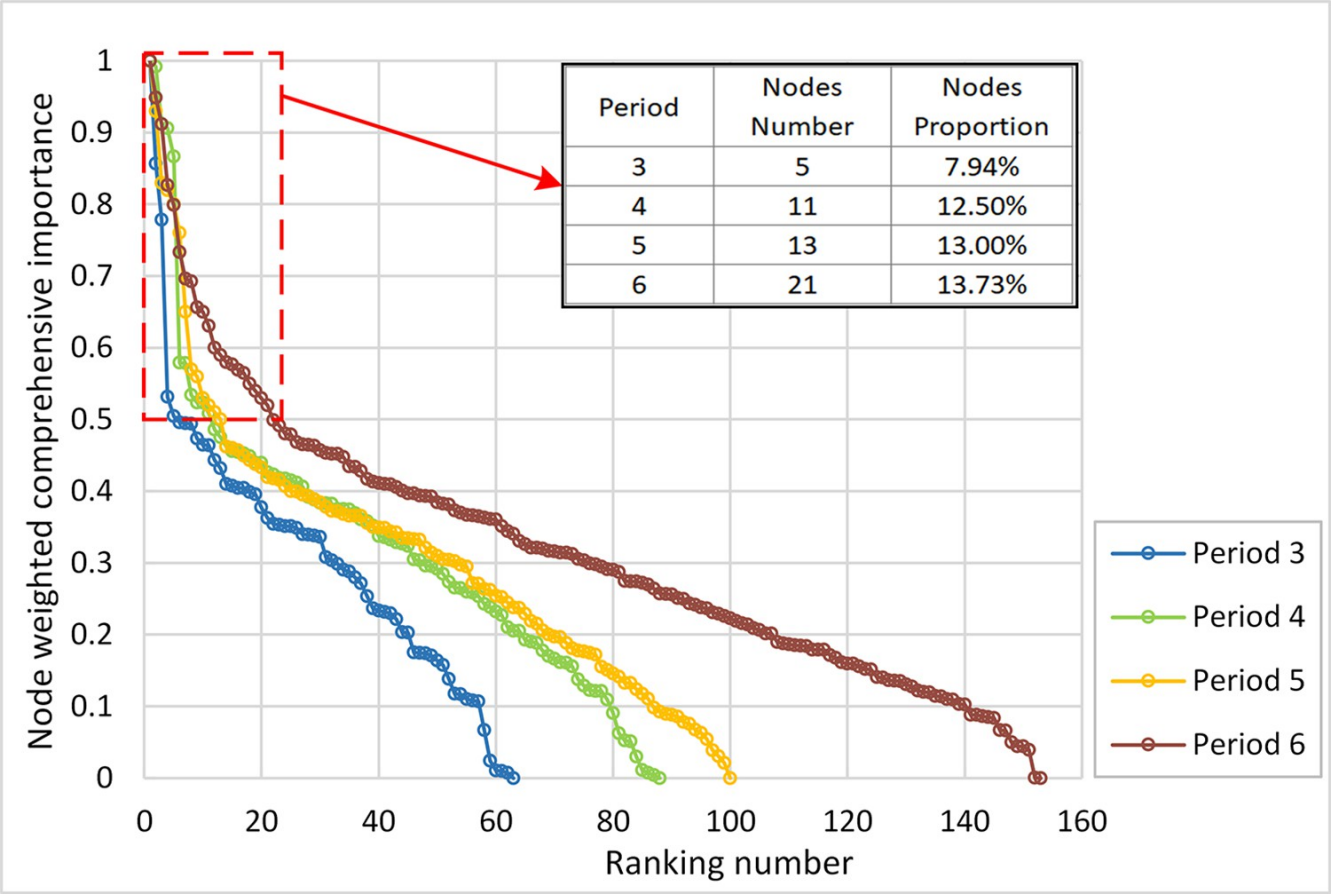
The node weighted importance decline curve, and the number and proportion of nodes with *WH*_*i*_ > 0.5.

The *WH*_*i*_ values of the nodes were normalized (*i*.*e*., values between 0 and 1) and ranked from largest to smallest. The ranking results were then connected in a line, as shown in Fig 5, where an inflection point is visualized in the node importance decline curve. This means that the nodes were divided into two categories according to their importance. Therefore, to ensure that the important nodes were not missed, the minimum value of inflection point (*WH*_*i*_ = 0.5) was considered the threshold value. The number and proportions of nodes with *WH*_*i*_ values greater than 0.5 were counted, referring to Fig 5. It is noteworthy that, after the network basic skeleton was formed in period 3, the number and proportion of important nodes steadily increased. This conclusion suggests that managers should scientifically judge the important nodes in different periods and pay full attention to potential important nodes in URTNs.

**Fig 6:**
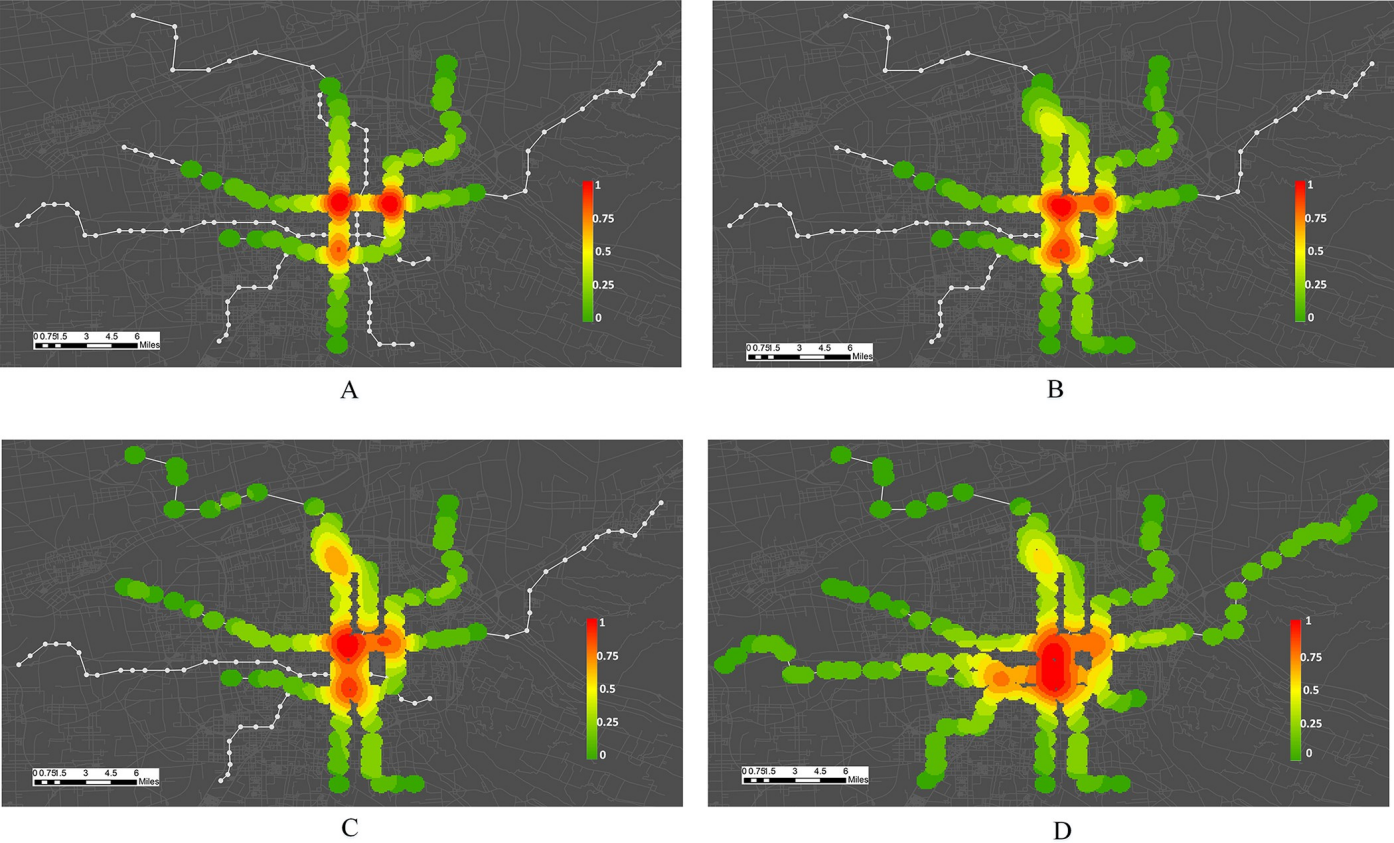
The distribution of the weighted comprehensive importance of nodes. A: Period 3, B: Period 4, C: Period 5, D: Period 6.

To reveal the spatial evolution of important nodes more clearly, the *WH*_*i*_ importance of the nodes was included on the map in Fig 6, where the colors closer to red indicate a higher importance of nodes. With the development of the XURTN, the spatial distribution of important nodes has undergone a dynamic redistribution. The importance of the different nodes was found to vary significantly in the same period; moreover, the importance of the same node varied significantly over time. In period 3, important nodes were concentrated in the line crossings. The stations in the parallel sections of *L-2* and *L-4* became important in period 4 as these nodes share the pressure on the north-south passenger corridor. With the opening of extension line, the important nodes in period 5 began to extend toward the airport. Finally, in period 6, the red and yellow areas area are connected into a sheet in the city center area and extend to the outer new districts.

This shows that the spatial migration of the important nodes is closely related to the new line types, the network evolution patterns and the direction of urban development. In the early stage of network development, important nodes are mainly concentrated at interchange stations in the city center. With the development of urban space and network, the important nodes are connected into pieces in the center area and gradually migrate to the peripheral new areas. After the opening of the peripheral lines, the importance of the connecting nodes between the peripheral branch lines and the central network increases significantly. This phenomenon indicates that these stations assume the connection between the central city and the new district in terms of structure and passenger flow, and furthermore, it also indicates that the lines and XURTN play a role in strengthening and facilitating the connectivity between different functional areas. It can be seen that the spatial migration of important nodes reflects the evolution of XURTN to some extent, which proves that the development of XURTN coincides with the trend of urban space towards the peripheral new districts.

Then, the differences between the important nodes identified by the single-factor methods, the topological comprehensive identification method and the *WH* method are compared, in order to demonstrate the superiority of *WH* method. The attributes of important nodes identified by the single-factor methods are relatively homogeneous, and most of them are interchange stations (e.g., nodes identified by degree, betweenness, and weighted betweenness) or stations with large passenger flow (e.g., nodes identified by strength, transportation reliability) or located in the center of the network (e.g., nodes identified by closeness, and weighted closeness). Moreover, single-factor identification methods have rough results. For example, node degree method considers most nodes to be of equal importance, and many nodes close to the edge of the network have the same betweenness and connectivity reliability. Most of the important nodes identified by the topological comprehensive identification method are located in the center network and transfer stations.

Comparatively, the important nodes, identified by the *WH* method, not only have large passenger flow (*e*.*g*., #11, #12 and #14) or are transfer stations (*e*.*g*., #10, #34 and #74), but they also include stations around the transfer stations (*e*.*g*., #30 and #36) and stations with small passenger flow (*e*.*g*., #48, #108 and #109), as well as the connections between the outer extended lines and the central region (e.g., #4, #36 and #48). It can be seen that the important nodes identified by the *WH* method have broader spatial distribution and more quantity and proportion, and the *WH* method can meticulously distinguish the importance of different nodes. Importantly, the *WH* method can obviously identify the trend of important nodes shifting to new areas, which cannot be identified by the single-factor and topological methods.

In summary, the *WH* method in the paper can identify the important nodes more comprehensively, detailed and objectively, as well as the evolution characteristics of the important nodes under the influence of multiple factors such as network structure, passenger flow, and urban space. More and more important nodes are extended to the peripheral new districts, reflecting to some extent the high degree of coincidence between XURTN and the development direction of urban space. These findings remind managers that adequate attention should be given to these established and potentially important nodes. In operation management, these important nodes are prioritized for protection and rehabilitation. Different protection and restoration levels are assigned to nodes. For example, the nodes in XURTN can be categorized into five levels, including special-grade nodes (WH ≥ 0.5), first-grade nodes (0.4 ≤ WH < 0.5), second-grade nodes (0.3 ≤ WH < 0.4), third-grade nodes (0.2 ≤ WH < 0.3), and fourth-grade nodes (WH ≤ 0.2). Nodes with high grade should be protected and repaired on priority and more defense resources should be allocated to improve the service level and security of URTN. When URTN planning and designing, it is significant to identify potential important nodes in advance and reserve the capacity of the stations and facilities, to avoid passenger congestion after the opening of new lines. From the viewpoint of the synergy and sustainable development between the URTN and the city, it should be ensured that the deployment of important nodes coincides with the spatial layout of the city, and high-intensity development is carried out around the stations relying on the accessibility of the important nodes, so that the URTN can play a role in guiding and promoting the city development.

### Comparison of resilience under different strategies

**Fig 7:**
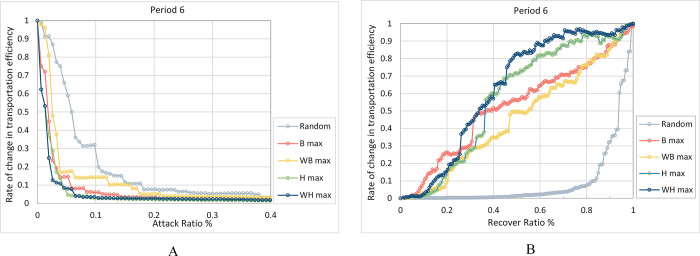
The change of network *E*(*G*^*w*^) under different strategies in period 6. A: Attack, B: Recover.

Based on the identification of the important nodes, five strategies are used to perform the simulations of attack and repair over XURTN. The experiments illustrate that the time complexity of all five strategies belonged to a square order *O*(*n*^2^), and the actual running time did not very much. Therefore, the network performance, after every node attack or repair, is plotted as a curve. Considering the period 6 as an example, Fig 7A shows the network performance for the first 40% of nodes attacked, when *F*(*t*) has dropped below 5%, and the damage index *R*_*dam*_ and the recovery index *R*_*rec*_ for each period are presented in Table 5.

**Table 5:** *R*_*dam*_ and *R*_*rec*_ values under different strategies during network evolution.

	Period	*WH max*	*H max*	*WB max*	*B max*	Random
***R***_***dam***_	**3**	0.0673	0.0723	0.0859	0.0837	0.0890
	**4**	0.0505	0.0521	0.0653	0.0592	0.1023
	**5**	0.0442	0.0449	0.0478	0.0505	0.0562
	**6**	0.0341	0.0345	0.0630	0.0437	0.0951
***R***_***rec***_	**3**	0.4472	0.4367	0.4210	0.4160	0.1496
	**4**	0.4634	0.4471	0.4556	0.3930	0.1567
	**5**	0.5366	0.5174	0.4804	0.4382	0.1087
	**6**	0.6385	0.5806	0.5299	0.4629	0.0970

Analyzed from a horizontal perspective, the network resilience, under different attack and repair strategies for the same period are compared. The results show that the network performance degradation or repair is faster under the malicious strategies compared to being exposed to the random strategy. Among the malicious strategies, the *WH max* strategy has the strongest effect on both the attack and the reparation of the network, as shown in the dark blue curve in Fig 7. In all periods, the *R*_*dam*_ variable, under the *WH max* attack strategy, represent the minimum values, which are 1.17%~178.89% smaller than other *R*_*dam*_ calculated when using the traditional strategies; moreover, the *R*_*rec*_ variable, under the *WH max* recovery strategy, represents the maximum values, which are 1.68%~84.81% larger than *R*_*rec*_ of other strategies. Finally, with the development of XURTN, the advantage of *WH max* strategy is becoming much more obvious.

This indicates that the degradation and repair of network performance is highly dependent on the node ranking, and a small number of important nodes plays a very important role in URTN. Importantly, the *WH* method is proved to be effective in improving the identification accuracy of important nodes, compared to the single-factor methods and the topological comprehensive importance method.

### Analysis of network resilience evolution results

**Fig 8:**
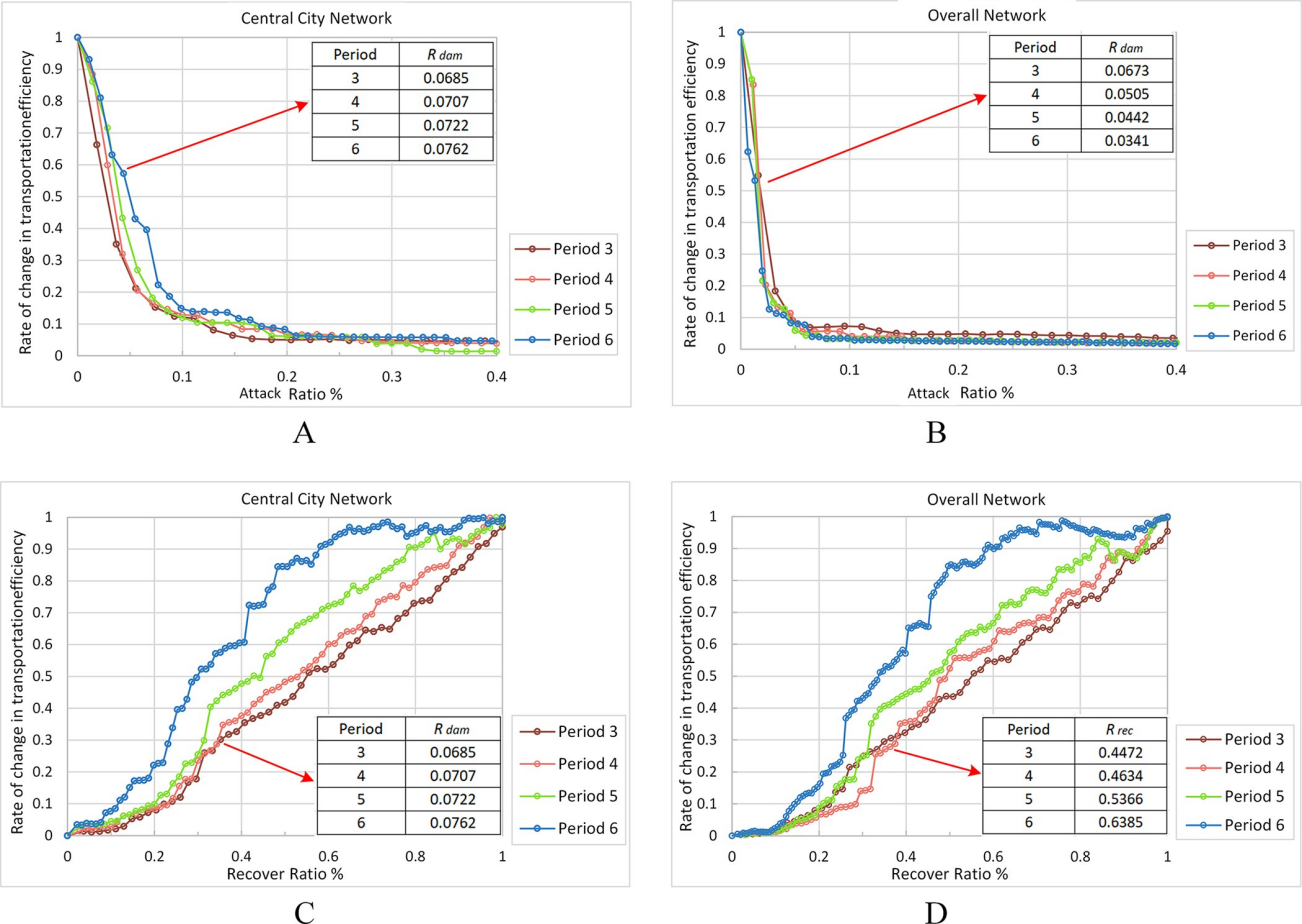
The change of network *E*(*G*^*w*^) under WH max strategy during network evolution. A: Attack central city network, B: Attack overall network, C: Recover central city network, D: Recover overall network.

Next, referring to the longitudinal perspective, the evolution of the network resilience during the growth of XURTN, under the *WH max* strategy, is analyzed emphatically. As the nodes are attacked sequentially, the *E*(*G*^*w*^) value declines rapidly. The dark blue line of Period 6 in Fig 8B decreases faster than the other lines, and the *R*_*dam*_ variable shows a decreasing trend from the period 3 to the period 6. It is clear that, with the development of XURTN, the overall network becomes gradually less resilient during the attack process. Moreover, the *WH max* attack simulation was carried out on the network in the central city (the central city area was shown in Fig 3), and the network performance degradation curve is shown in Fig 8A. The *R*_*dam*_ value of the central city network is significantly higher than that of the overall network, and it gradually increases with the network growth.

The restoration of the central city network and the overall network, under the *WH max* strategy, are shown in Fig 8C and 8D. As the network grows, its performance is repaired much faster, and the central city network gets a better restoration capability than the overall network. This indicates that, with the growth of XURTN, the overall network restoration capability gradually increases; however, the peripheral lines reduce the restoration capability.

It can be shown that longer peripheral lines negatively affect the resilience of XURTN in both the attack and the repair progresses. In particular, during the period 5 and period 6, the *R*_*dam*_ value of the central city network increased by 63.35% and 123.46% compared to the overall network, whereas the *R*_*rec*_ variable increased by 11.65% and 5.12%, respectively. The fundamental reason for declining the XURTN resilience is that the network structure has flaws.

As the network grows, there is a desire to build lines as to compensate for the current network’s shortcomings. However, some new lines do not provide positive improvements in some emergency cases, even though they increase the spatial coverage of the network. When the stations, in the central area, are disrupted, passengers can choose alternative routes to reach their destinations because the central network provides multiple alternative routes. However, when the nodes at the periphery or the connection between the center and the peripheral area fail, the passengers do not have alternative route. If the new line is not sufficiently connected to the central network, the failure of the nodes at the connection will lead to a disconnection between the peripheral lines and the central network, which may lead to the failure of the whole line. Therefore, as the URTNs grow, the number of peripheral lines gradually increases as well as the important nodes. Thus, the removal of these important nodes will significantly degrade the network performance. In this case, the new lines have a negative impact on the resilience of the network.

Therefore, when planning the network, the construction of the central lines should be accelerated to meet the huge traffic demand in the central city. At the same time, the lines that radiate to the key new areas should be encrypted, in order to achieve fast and convenient access between the central area and the new area and between the key new areas. And it should strengthen the connection of peripheral lines to the central network and effectively increase the redundancy provided by these lines, in order to ensure their positive effectiveness on network connectivity and resilience. Moreover, when managing operations, it is important to strengthen the protection of not only the stations in the central area, but also some important stations on the extension line, which may cause serious loss of network performance.

## Conclusion

To study the evolution of the dynamic resilience of URTNs with their development, a dynamic resilience assessment model, based on a ridership-weighted network, was proposed in this work. The model comprehensively analyzed the full cycle of emergency occurrence and repair, and introduced the network transport effectiveness *E*(*G*^*w*^), to quantitatively assess the network resilience. Moreover, the node comprehensive importance identification *WH* method was proposed and implemented in this paper. Then, XURTN 2011–2021 was considered as the case study to analyze the evolutionary characteristics of the network resilience.

In terms of important nodes evolution, the *WH* method integrates multiple factors, such as network structure and passenger flow, to comprehensively and meticulously identify the important nodes in the URTN, and discovers the evolutionary characteristics of the important nodes. After the basic skeleton of the XURTN is formed, the number and proportion of the important nodes continue to grow. The spatial evolution direction of the important nodes is highly correlated with the type of newly opened lines. The more and more important nodes are migrating to the peripheral new districts, which to some extent reflects the synergistic evolution of the network and the urban space. Notably, the node identified by the *WH* method had a stronger effect on the destruction and repair of the network, resulting in the *R*_*dam*_ under the *WH max* strategy that was 1.17%~178.89% smaller and the *R*_*rec*_ that was 1.68%~84.81% larger than those obtained when using the single-factor and topological strategies. Therefore, it is proved that the *WH* method improves the accuracy of the important node identification.

In terms of the evolution of the resilience, XURTN has formed a single-center network structure, containing a dense center and several longer-length branch structures, extending therefore the periphery accessibility. As the network evolves, the overall network resilience in the attack progress is gradually decreasing, while the resilience in the recovery progress is gradually increasing. The network structure and the distribution of important nodes are found to have significant impacts on the network resilience. Although the new lines improve the spatial accessibility, the longer peripheral lines affect negatively the resilience of XURTN due to the inadequate connection of the peripheral lines to the central area. Especially in the simulated attack process, the resilience of the overall network is gradually weakening with the development of XURTN, while the resilience of the central city network is gradually increasing. For example, during the period 5 and period 6, the *R*_*dam*_ of the central city network increase by 63.35% and 123.46%, respectively, compared to the overall network, where the *R*_*rec*_ value increase by 11.65% and 5.12%, respectively.

In summary, the city is an evolving living organism with expanding spatial scales and growing peripheral new areas. Consequently, the transportation needs are constantly changing, especially the demand within the new peripheral areas and the demand between the central area and the peripheral areas are constantly increasing. The URTN should play a positive role in undertaking, guiding, and facilitating the urban space and travel demand, so the planning and operation management of the network is of vital importance. Based on the conclusions of this paper, recommendations for network improvement are presented in the following aspects:

1. Enhancing the identification of important nodes and adopt different levels of protection and restoration strategies for nodes.
  With the development of URTNs, stations, that were not important in the early period of this network formation, may become important after the opening of new lines. For example, after the opening of L-5 in Xi’an, Nanshaomen station experienced serious platform congestion and queuing. Therefore, the management should strengthen the prediction and protection of potentially important nodes, and shift from passive repair to the active protection. Specifically, managers should classify nodes into different levels of protection and restoration, enhance monitoring and protection of important stations and key equipment, and formulate detailed and reliable emergency rescue plans.
2. Enhancing and optimizing the connection between peripheral and urban lines, and promoting the connection and development of different urban areas.
  When planning and optimizing the network, the connection between peripheral and central-city lines should be increased, and the layout of interchange stations should be reasonably optimized, so that there will be a greater number of important nodes in the network with a more balanced spatial distribution. On the one hand, it helps to improve the network structure and the uniformity of passenger flow, and the resilience of the network. On the other hand, it makes the URTN better promote the connection of different urban areas, guide the development of the city to the peripheral new areas, and optimize the urban spatial pattern.
3. Constructing the integrated development of urban rail transit and multiple modes of public transportation.
  Within a certain area, the synergistic development of urban rail transit and ground public transportation should be constructed. When an emergency occurs in an urban rail transit, passengers can be diverted to ground buses for transfer, in order to enhance the resilience of URTN. In addition, with the gradual expansion of the urban spatial scale, different modes of rail transit with different service functions should be deployed in different scales of space, such as intercity, suburban and urban rail transit. Realizing the multi-network integration development of rail transit helps to support the spatial pattern of urban polycentricity.

However, this study still has some limitations. First, to simplify the calculation, assumptions were made about the loss and return of passenger flow when simulating network failure and repair. Therefore, in future research, the change process of passenger flow should be optimized by combining the travel characteristics of passengers in real scenarios. Second, different node importance metrics were considered, and the weights of each metric were calculated linearly. However, it is necessary to improve the selection of the importance metrics as well as the weight determination methods, and the multi-attribute decision method. Finally, the network resilience in the morning and evening peak periods can be further explored, which can help to promptly control the network.

## Supporting information

S1 File (DOCX)
