# Supplementary material for: Assessing the dynamic resilience of Urban Rail Transit Networks during their evolution using a ridership-weighted network
Source: PLoS One. 2023 Sep 21;18(9):e0291639. doi: 10.1371/journal.pone.0291639 (PMC10513224; doi:10.1371/journal.pone.0291639)
Supplement: S1 File — (DOCX) [file pone.0291639.s001.docx]

# Notations

As a convenient reference, the mathematical notations used in this paper are presented below.

| *G* | An undirected and unweighted network | *E(G^w^)* | Network transport efficiency |
| --- | --- | --- | --- |
| *G^w^* | A directed and weighted network | *Q_ij_* | Passenger flow between *i* and *j* |
| *V* | The nodes set | *$E(G_{t}^{w})$* | Network transport efficiency at time *t* |
| *V_i_* | The *i^th^* station in network | *$E(G_{0}^{w})$* | Network transport efficiency in normal state |
| *E* | The edges set | *K_i_* | Degree of node *i* |
| *e_ij_* | The edge between *i* and *j* | *S_i_* | Strength of node *i* |
| $E^{w}$ | The directed edge set | *B_i_* | Betweenness of node *i* |
| $e_{ij}^{w}$ | The directed edge between *i* and *j* | *$\sum_{o\neq d\neq i} n_{od}^{'}$* | The number of shortest paths through i between o and d |
| *W* | Weights set | *$\sum_{o\neq d} n_{od}$* | The number of shortest paths between o and d. |
| ${pf}_{e_{ij}^{w}}\left( \Delta t \right)$ | Weight of the directed edge $e_{ij}^{w}$ | *WB_i_* | Weighted betweenness of node *i* |
| *N* | Number of nodes | *$w_{l}^{x}$* | The weight of the *x^th^* edge on the lth path between *ij* |
| *M* | Number of edges | *$w_{l}^{i,x}$* | The weight of the *x^th^* edge on the lth path passing through *i* |
| *N_L_* | Number of lines | *n* | The number of shortest paths in the network |
| *L* | Total length of network | *n^i^* | The number of these shortest paths passing through *i*. |
| *N_C_* | Number of transfer stations | *C_i_* | Closeness of node *i* |
| *N_P_* | Proportion of transfer stations | *WC_i_* | Weighted closeness of node *i* |
| *PF* | Average daily passenger flow volume of URTN | *P_i_* | The node connectivity performance |
| *β* | The complexity | *PG* | The number of connectable node pairs in *G* |
| *α* | The availability of loops | *PG＇* | The number of connectable node pairs after *i* is attacked. |
| *λ* | The connectivity | *F_i_* | The node transportation performance |
| *d_ij_* | The shortest path length between *i* and *j* | *F* | The sum of all cross-sectional flows in the network during normal operation. |
| *$\text{d}_{ij}^{w}$* | The weighted shortest path length between *i* and *j* | $F_{i}^{'}$ | The sum of all cross-sectional flows after *i* is attacked. |
| $F(t)$ | Network performance function | *H_i_* | The comprehensive importance of node *i* |
| $R_{dam}$ | The damage index | ${WH}_{i}$ | The weighted comprehensive importance of node *i* |
| $R_{rec}$ | The recovery index | *μ, δ* | Weight coefficient of node importance indexes |
| *E(G)* | Network efficiency |  |  |
